# Prefrontal Cortical Asymmetry and Motor Slowing in Older Women: EEG Evidence That Fear of Falling Modulates Emotional Valence and Reaction Time

**DOI:** 10.1111/psyg.70174

**Published:** 2026-05-06

**Authors:** Guilherme Augusto Santos Bueno, Murielle Celestino da Costa, Katarine Souza Costa, Renato Canevari Dutra da Silva, Elton Brás Camargo Júnior, Germano Gabriel Lima Esteves, Ruth Losada de Menezes

**Affiliations:** ^1^ Department of Medicine University of Rio Verde Goiás GO Brazil; ^2^ Postgraduate Program in Health Sciences and Technologies, University of Brasília Federal District DF Brazil; ^3^ Graduate Program in Health Sciences, Faculty of Medicine, Interdisciplinary Center on Aging, Federal University of Goiás Goiânia Brazil; ^4^ Department of Odontology University of Rio Verde Goiás GO Brazil; ^5^ Department of Postgraduate University of Rio Verde Goiás GO Brazil; ^6^ Department of Psychology University of Rio Verde Goiás GO Brazil

## Abstract

**Aim:**

To investigate the relationship between cortical activation and motor performance in older women with different levels of fear of falling (FoF) and fall history.

**Methods:**

Fifty‐five participants were evaluated, including 40 older adults divided into four groups (NotFall‐LFoF, NotFall‐HFoF, Fall‐LFoF, Fall‐HFoF) and 15 younger controls. Motor reaction time was assessed using adapted TRT_S2012 software, while cortical activity was recorded via EEG (EMOTIV EPOC+). Cortical arousal was indexed by the β/α ratio, and valence by (αF4/βF4) − (αF3/βF3) asymmetry. Statistical analyses included ANOVA and Pearson's correlation (α ≤ 0.05).

**Results:**

Groups were homogeneous in demographic and cognitive characteristics. Significant differences were observed in cortical arousal (*p* = 0.014) and valence (*p* = 0.004). Higher FoF levels were associated with reduced prefrontal symmetry and slower reaction times. Strong negative correlations were found between valence and reaction times (*r* > −0.9). FES‐I scores showed positive correlations with motor latency (*r* = 0.8–0.9) and negative correlations with cortical indices (*r* = −0.7 to −0.9).

**Conclusions:**

Fear of falling modulates prefrontal cortical activation, shifting motor control from automatic to more conscious processing, which impairs motor efficiency. FoF emerges as a potential cortical biomarker of motor vulnerability, reinforcing the importance of neurorehabilitation strategies integrating emotional and cortical regulation to improve mobility and reduce fall risk in aging populations.

## 1 Introduction

Falls represent one of the most disabling clinical events of aging, resulting in injuries, loss of independence, and increased mortality [1, 2]. Although frequent, falls are not part of the physiological course of aging but rather result from the interaction between intrinsic factors such as deficits in motor control, muscle strength, and sensorimotor integration and extrinsic factors, including environmental hazards and drug‐related iatrogenesis. Consequently, falls are considered a marker of functional and cognitive vulnerability, reflecting fragility in postural and motor control systems [3, 4, 5].

Over recent decades, understanding of fall mechanisms has expanded beyond the musculoskeletal domain to include cognitive and emotional dimensions. Functions such as attention, decision‐making, and inhibitory control contribute to gait and balance regulation, while affective factors such as fear of falling (FoF) modulate motor behaviour and the risk of future falls [6, 7, 8]. This conceptual integration has driven the development of multidisciplinary prevention strategies that combine physical, cognitive, and emotional approaches.

Fear of falling has emerged as one of the most relevant and paradoxical psychogenic factors. Although it initially serves a protective purpose, FoF often persists even in the absence of prior falls, negatively influencing body perception and motor control [7, 9]. Fear alters gait automaticity and fluency, leading to segmental rigidity and movement slowing, paradoxically increasing the likelihood of future falls [10, 11]. Previous findings also suggest that FoF predicts motor alterations more strongly than an actual history of falls [10, 12].

Recent neuroscientific evidence indicates that FoF is not merely a behavioural phenomenon but rather a neuroemotional state mediated by prefrontal circuits [7, 9, 10]. The prefrontal cortex (PFC) plays a central role in fear and anxiety regulation, modulating emotional and motor responses [13, 14]. Hyperactivity of the right and orbitofrontal PFC, observed in older women with high fear levels, reflects increased cognitive involvement in movement control, resulting in greater attentional cost and slower responses [14, 15, 16].

During aging, compensatory neural patterns emerge, characterized by enhanced activation of motor and somatosensory cortical regions, as demonstrated by functional neuroimaging and near‐infrared spectroscopy studies [17, 18, 19, 20, 21]. Although adaptive, this increased cortical recruitment is associated with loss of motor automaticity and greater reliance on cognitive networks [22].

Electroencephalography (EEG) provides a sensitive, real‐time measure of these cortical alterations. Oscillations in the alpha (8–12 Hz) and beta (13–30 Hz) bands are recognized as indicators of cortical state, with the beta/alpha ratio reflecting neural arousal and the frontal asymmetry (F3–F4) index representing emotional valence [23, 24]. Right‐hemispheric dominance, resulting in reduced valence, has been associated with anxiety and avoidance states and correlates with motor slowing and longer reaction times [25, 26, 27].

Within this framework, understanding how FoF influences cortical activity and reaction time in older women may reveal subtle physiological mechanisms that precede falls, enabling more precise, cortex‐cantered prevention strategies. Therefore, this study aimed to assess prefrontal cortical activity (valence and arousal) and motor performance (simple and fatigue reaction times) in older women with varying levels of FoF and fall history, using EEG. The central hypothesis was that FoF acts as a cortical modulator of movement, manifested by reduced prefrontal valence and increased motor latency, even in the absence of structural or cognitive deficits.

## 2 Materials and Methods

### 2.1 Study Design and Ethics

This was a cross‐sectional and analytical study conducted in a controlled laboratory environment. The protocol was approved by the Research Ethics Committee of the University of Brasília, Faculty of Ceilândia (approval number 2.109.807). All procedures were performed in accordance with the principles of the Declaration of Helsinki. Participants were informed about the study objectives and signed an informed consent form prior to participation.

### 2.2 Study Population

Participants were invited to take part in the research and were screened for eligibility before enrollment. The inclusion criteria were: (i) female sex; (ii) age ≥ 65 years; (iii) ability to ambulate independently in the community without walking aids; (iv) absence of previous surgeries on the lower limbs, pelvis or spine; (v) body mass index (BMI) < 28 kg/m^2^ [28]; (vi) preserved cognition, assessed by the Mini‐Mental State Examination (MMSE) [29], with a minimum score of 18 adjusted for education level [30]; (vii) absence of medical diagnosis of rheumatoid arthritis, neuromuscular or neurodegenerative disease, including diabetes mellitus; (viii) absence of visual impairment; (ix) abstinence from alcohol intake in the 24 h preceding data collection; and (x) no prior contact with the instrumented gait analysis laboratory.

All eligible participants were instructed about the procedures and signed the informed consent form.

The sample size was estimated using G*Power 3.1.9.2 software (Franz Faul, Universität Kiel, Germany) [31], based on a one‐way analysis of variance (ANOVA) for simple reaction time. A total sample of 40 older women (*n* = 10 per group) was required to detect a statistically and clinically significant difference related to exposure to fear of falling, considering an effect size (*ω* ^2^) of 0.82, *α* = 0.05 and power = 0.99. An additional 15 younger adult women were included to compose the control group.

### 2.3 Measurements and Definitions

Participants were classified according to fall history and fear of falling (FoF), resulting in two experimental groups of older women (fallers and non‐fallers) and one control group of younger women. The experimental sample was further divided into four subgroups: fallers with low fear of falling (Fall‐LFOF), fallers with high fear of falling (Fall‐HFOF), non‐fallers with low fear of falling (NotFall‐LFOF) and non‐fallers with high fear of falling (NotFall‐HFOF).

Fall history was determined based on the definition proposed by Lamb, Ellen and Hauer (2005) [32], as “an unexpected event in which the participant comes to rest on the ground, floor or a lower level.” The presence of at least one fall in the past 12 months was used as a criterion, producing a dichotomous classification of “faller” or “non‐faller.”

Fear of falling was assessed using the Falls Efficacy Scale–International (FES‐I) [33], in its Brazilian cross‐cultural validation [34]. The instrument evaluates concern about falling during 16 daily activities, rated on a 4‐point scale ranging from 1 (“not at all concerned”) to 4 (“very concerned”), with total scores ranging from 16 to 64. Based on previous studies, participants were classified as having low fear of falling (≤ 27) or high fear of falling (> 27) [35].

### 2.4 Motor Reaction Time Assessment

Motor reaction time and coordination were assessed using the TRT_S2012 software, adapted with a pedal‐type joystick as the response interface. This device was specifically designed to capture reaction time at the level of the lower limb, given that neuromusculoskeletal mechanisms associated with fear of falling are more directly related to lower limb function, as demonstrated in gait and motor control studies, including previous work from our research group [36].

Participants were seated in a stable chair to ensure safety and eliminate balance demands. The feet were initially positioned in a neutral position over the pedal. Upon visual stimulus presentation, participants were instructed to perform a slight plantarflexion movement (~10°–15°) to press the pedal. The device had minimal mechanical resistance, ensuring that the response primarily reflected reaction time rather than strength or power.

Standardized instructions from the TRT protocol were followed, in which participants were asked to respond as quickly as possible to the visual stimulus, without emphasis on force or urgency beyond normal response execution. Given the seated position and the absence of postural or locomotor demands, the task did not impose balance challenges or induce instability. This setup was designed to minimize the influence of fear of falling on motor execution, ensuring that differences in reaction time were more closely related to cortical processing and motor response speed rather than behavioural caution or postural threat.

**FIGURE 1:**
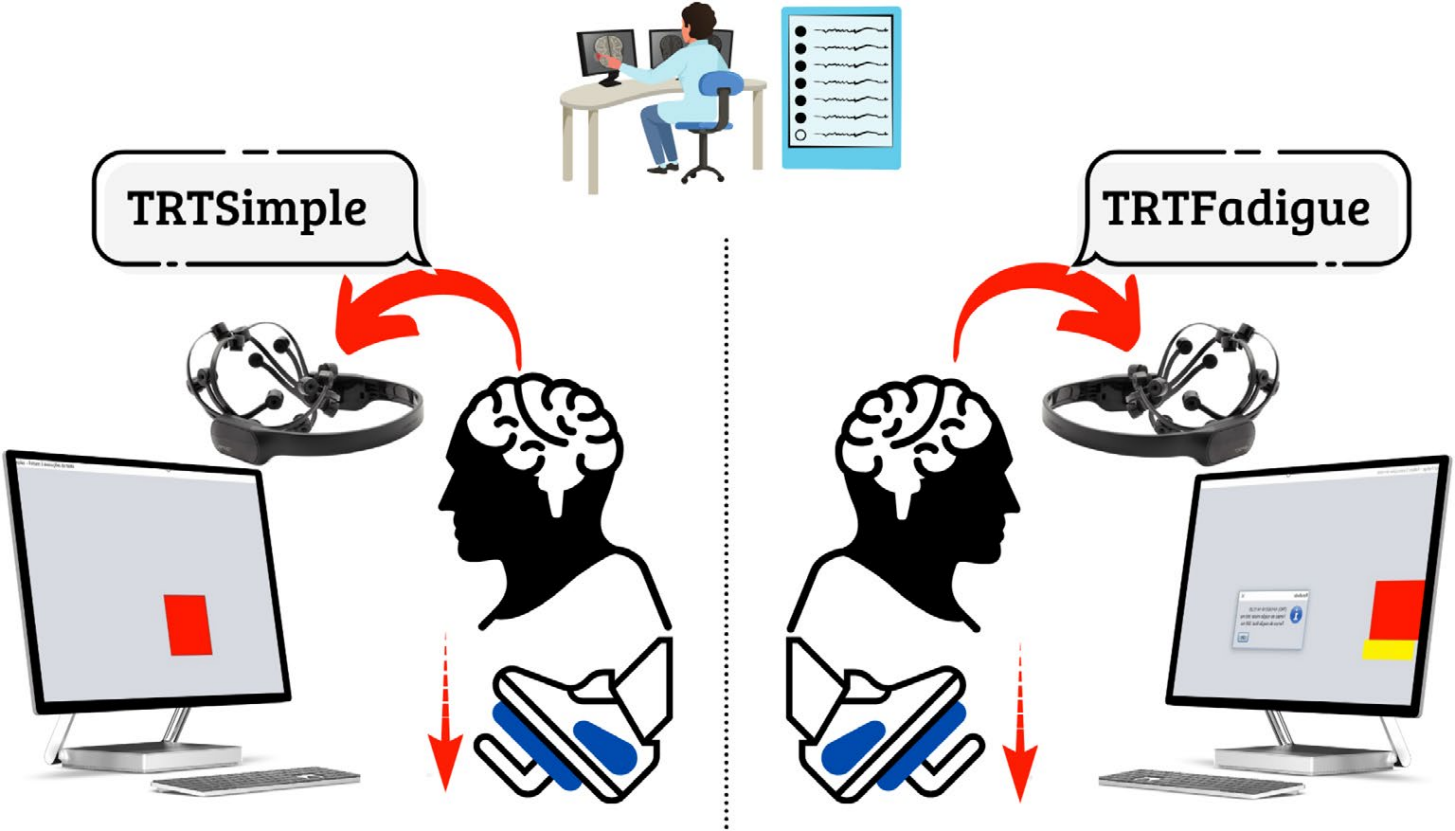
Motor reaction time assessment combined with electroencephalography. *Source:* Author.

In the Simple Reaction Time Test (TRT‐Simple), a red square appeared at the centre of the monitor at predetermined intervals ranging from 1.5 to 6.5 s, with randomized timing between trials. Upon the appearance of the stimulus, participants were required to respond as quickly as possible by pressing the pedal joystick (Figure 1).

The Fatigue Reaction Time Test (TRT‐Fatigue) involved following the movement of a red bar displayed on the screen, shifting horizontally from left to right. Participants were instructed to press the pedal joystick immediately when the red bar appeared and to keep it pressed while the bar moved across the screen. The button was to be released precisely when the red bar disappeared. Two variables were recorded: TRTi‐Fatigue, corresponding to the pressing latency, and TRTf‐Fatigue, corresponding to the release latency [36].

The software protocol included two familiarization trials, followed by five TRT‐Simple trials and a sequence of TRT‐Fatigue trials.

### 2.5 Electroencephalography (EEG) Recording and Processing

**FIGURE 2:**
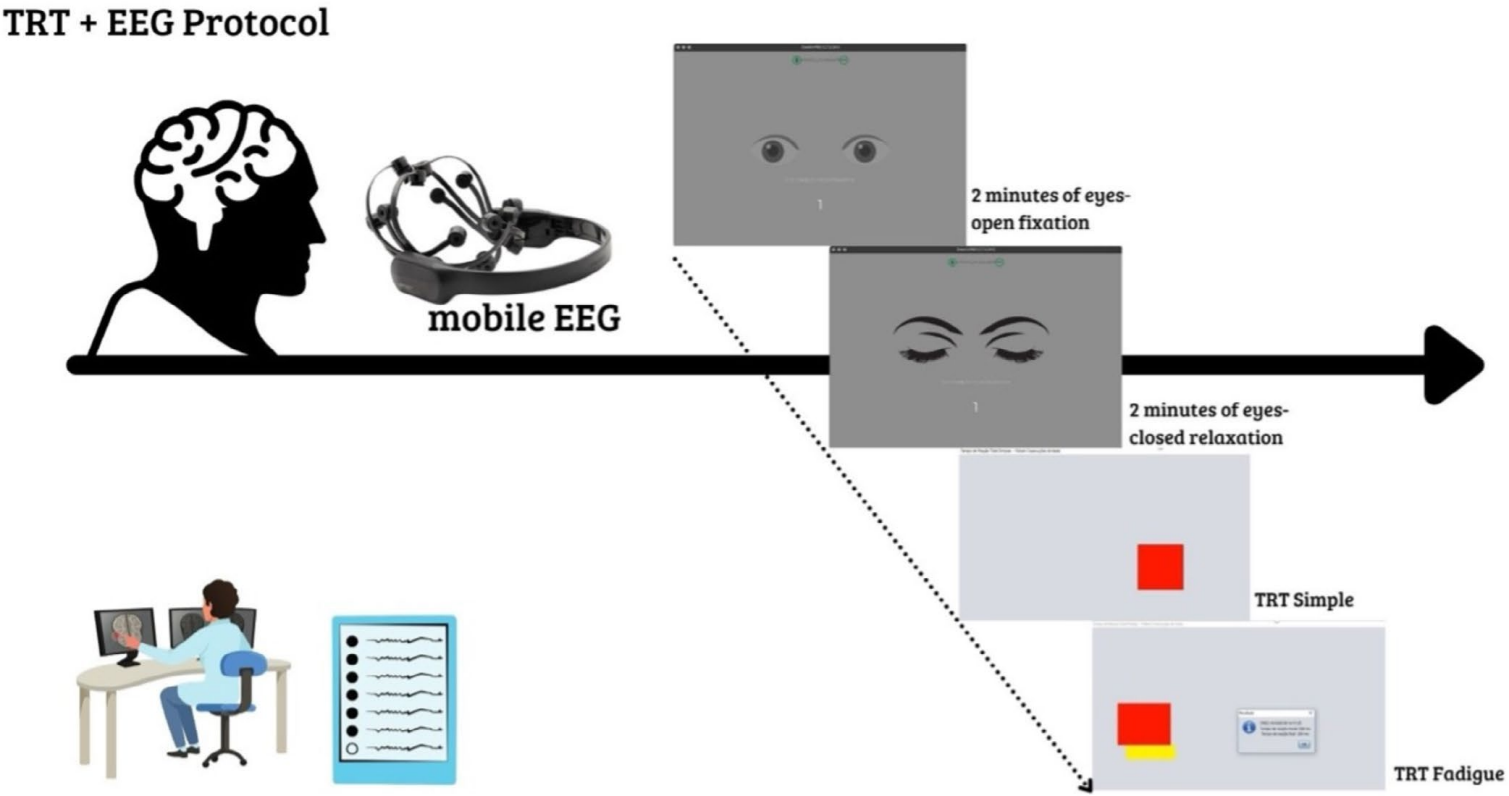
EEG preparation protocol for the motor reaction time task. *Source:* Author.

In this study, electroencephalographic signals were recorded using the EMOTIV EPOC+ system (Emotiv Inc., San Francisco, USA) [37, 38], as illustrated in Figure 2. The EMOTIV EPOC+ is a high‐resolution, portable EEG system equipped with 14 active data acquisition electrodes (AF3, F7, F3, FC5, T7, P7, O1, O2, P8, T8, FC6, F4, F8 and AF4) and two reference electrodes (P3 and P4). Several studies have validated this system for scientific research applications [39, 40, 41]. EEG enables real‐time monitoring of neural activity on a millisecond scale and provides a highly sensitive measure for detecting subtle differences in neural oscillations [42].

EEG signals were continuously recorded during the motor reaction time tasks. Data acquisition was temporally synchronized with the TRT_S2012 software, ensuring alignment between neural activity and task execution (Figure 1). Specifically, EEG recording windows corresponded to the interval between the visual stimulus presentation on the screen and the participant's motor response. This approach allowed the analysis of cortical activity encompassing both anticipatory processing and motor execution phases, providing a temporally integrated measure of prefrontal engagement during task performance.

To extract relevant neural features, preprocessing of the raw EEG data was performed to remove noise and trivial information. Offline analysis was carried out using the EEGLAB toolbox [43] in MATLAB R2019b (The MathWorks, Natick, MA, USA). The preprocessing pipeline included downsampling the signal to 250 Hz and applying a 0.01–45 Hz Butterworth filter. The Independent Component Analysis (ICA) algorithm was used to identify and remove artefacts [44, 45].

Artefact‐related components were manually inspected for each participant and excluded based on their energy spectra, topographic distribution, and visual characteristics prior to inverse ICA reconstruction. On average, a small number of components per participant were removed, consistent with standard EEG preprocessing practices. Analyses were conducted considering EEG segments corresponding to the interval between visual stimulus presentation in the TRT_S2012 and the participant's motor response. After preprocessing, the level of residual artefacts within these segments was minimal, and no systematic differences were observed across groups, indicating comparable signal quality for cortical analyses. The cleaned datasets were then filtered into the conventional EEG frequency bands: δ (0.5–4 Hz), θ (4–8 Hz), α (8–13 Hz), β (13–30 Hz) and γ (30–45 Hz), as recommended by Stam and De Bruin (2004) [46].

Cortical arousal levels were determined by calculating the ratio of beta (12–28 Hz) to alpha (8–12 Hz) power. EEG signals were extracted from four prefrontal electrode sites (AF3, AF4, F3 and F4). Beta activity (β) is typically associated with alertness and engagement, while alpha activity (α) is dominant during relaxed or idle states. The beta‐to‐alpha ratio is therefore considered a reliable index of cortical arousal [47]. and was computed as follows:

$$\begin{array}{cc} \text{Arousal level}=\; & \left(\mathrm{ΒF}3+\mathrm{ΒF}4+\mathrm{ΒAF}3+\text{ΒAF4}\right)/\\ & \left(\mathrm{\alpha F}3+\mathrm{\alpha F}4+\text{αAF3}+\text{αAF4}\right) \end{array}$$

Cortical valence was determined by comparing the relative activation of the two hemispheres. Numerous EEG studies [48, 49, 50] have demonstrated that the left frontal area is associated with positive affect and memory, whereas the right frontal region is more involved in negative emotions. The F3 and F4 electrodes were selected because they are widely used to assess prefrontal alpha/beta asymmetry, a reliable index of emotional valence. Valence values were calculated by comparing the alpha and beta power between the left and right hemispheres [47], using the following formula:

$$\text{Valence}=\left(\mathrm{\alpha F}4/\text{βF4}\right)-\left(\mathrm{\alpha F}3/\text{βF3}\right)$$

### 2.6 Covariates

Potential confounding factors were controlled for, including age, sex, body weight, height, and body mass index. Additional covariates known to be associated with both fall risk and fear of falling were also evaluated, namely cognitive level [51]; and fall history [51, 52, 53].

### 2.7 Statistical Analysis

All analyses were performed using SPSS version 23.0 (IBM Corp., Armonk, NY, USA). The Shapiro–Wilk test was used to verify data normality. Continuous variables were expressed as mean ± standard deviation (SD). Group comparisons were conducted using one‐way analysis of variance (ANOVA) followed by Tukey's post hoc test. Effect sizes were calculated using *ω* ^2^ and *r*. To control for multiple comparisons, Bonferroni correction was applied where appropriate, as confirmed by an independent statistical review. The relationships between reaction time and cortical measures were examined using Pearson's correlation coefficient, interpreted as weak (*r* ≤ 0.30), moderate (0.31–0.69), or strong (*r* ≥ 0.70). The level of statistical significance was set at *p* ≤ 0.05.

## 3 Results

A total of 55 participants were included, consisting of one control group of younger women and four experimental groups of older adults classified by fall history and fear of falling: non‐fallers with low fear (NotFall‐LFOF), non‐fallers with high fear (NotFall‐HFOF), fallers with low fear (Fall‐LFOF), and fallers with high fear (Fall‐HFOF). Groups were homogeneous in weight, height, and cognitive status (*p* > 0.05). However, there were significant differences in age (*p* < 0.001) and body mass index (BMI) (*p* = 0.007), with higher BMI among older participants. Fear of falling, measured by the FES‐I Brazil, differed markedly among groups (*p* < 0.001; *ω* = 0.56), being higher in NotFall‐HFOF and Fall‐HFOF groups (34.3 ± 5.9 and 32.2 ± 4.5) compared with controls (20.6 ± 3.7) (Table 1).

**TABLE 1:** Sociodemographic characteristics, cognitive status, and fear of falling comparisons among experimental groups and the control group.

		Mean ± SD	Confidence interval (CI)	*p*‐value (*ω*)	Pairwise comparison									
					A/B (r)	A/C (r)	A/D (r)	A/E (r)	B/C (r)	B/D (r)	B/E (r)	C/D (r)	C/E (r)	D/E (r)
Age (years)	Control group	24.81 ± 6.82	18.21–29.21	< 0.001 (0.47)	—	—	—	< 0.001 (0.68)	—	—	< 0.001 (0.64)	—	< 0.001 (0.71)	< 0.001 (0.71)
	NotFall‐LFOF	72.50 ± 6.04	68.66–76.34											
	NotFall‐HFOF	72.67 ± 7.59	68.46–76.87											
	Fall‐LFOF	70.83 ± 5.59	67.28–74.38											
	Fall‐HFOF	73.90 ± 6.56	69.21–78.59											
Body weight (Kg)	Control group	55.68 ± 6.62	48.31–61.02	0.530 (−0.04)	—	—	—	—	—	—	—	—	—	—
	NotFall‐LFOF	61.61 ± 6.37	57.56–65.66											
	NotFall‐HFOF	58.05 ± 5.03	52.50–63.66											
	Fall‐LFOF	60.53 ± 7.78	54.98–66.07											
	Fall‐HFOF	63.27 ± 6.62	54.96–71.58											
Height (m)	Control group	1.59 ± 0.04	1.54–1.67	0.154 (−0.06)	—	—	—	—	—	—	—	—	—	—
	NotFall‐LFOF	1.55 ± 0.05	1.52–1.59											
	NotFall‐HFOF	1.54 ± 0.05	1.51–1.56											
	Fall‐LFOF	1.56 ± 0.08	1.51–1.60											
	Fall‐HFOF	1.53 ± 0.06	1.48–1.57											
Body mass index (kg/m^2^)	Control group	20.83 ± 2.28	19.45–23.20	0.007 (0.12)	—	—	—	0.019 (0.25)	—	—	0.035 (0.18)	—	—	—
	NotFall‐LFOF	25.57 ± 2.65	23.88–27.25											
	NotFall‐HFOF	24.67 ± 4.53	22.16–27.18											
	Fall‐LFOF	24.91 ± 2.34	23.42–26.39											
	Fall‐HFOF	27.04 ± 3.47	24.20–29.88											
Mini‐Mental State Examination (score)	Control group	25.65 ± 3.46	22.72–28.52	0.882 (−0.07)	—	—	—	—	—	—	—	—	—	—
	NotFall‐LFOF	26.50 ± 3.15	24.50–28.50											
	NotFall‐HFOF	26.93 ± 2.49	25.55–28.31											
	Fall‐LFOF	25.00 ± 3.19	22.97–27.03											
	Fall‐HFOF	27.70 ± 2.87	25.65–29.75											
FES‐I (score)	Control group	20.63 ± 3.74	17.50–20.14	< 0.001 (0.56)	< 0.001 (0.77)	—	< 0.001 (0.77)	—	< 0.001 (0.75)	—	< 0.001 (0.79)	< 0.001 (0.75)	—	< 0.001 (0.72)
	NotFall‐LFOF	22.33 ± 3.87	19.88–24.79											
	NotFall‐HFOF	34.27 ± 5.87	31.01–37.52											
	Fall‐LFOF	23.17 ± 3.74	20.79–25.54											
	Fall‐HFOF	32.20 ± 4.49	28.99–35.41											

*Note:* A = NotFall‐LFoF; B = NotFall‐HFoF; C = Fall‐LFoF; D = Fall‐HFoF. Comparative analysis was performed using one‐way ANOVA, considering effect size (*ω*) and significance at *α* ≤ 0.05. Post hoc comparisons were conducted using Tukey's test, with effect size (*r*) and significance set at *α* ≤ 0.05.

For motor performance, significant group differences were found in simple reaction time (TRTSimple) (*p* < 0.001; *ω* = 0.17). Controls exhibited faster responses (352.18 ± 98.21 ms) than older women, whose mean latencies increased progressively according to fear of falling: 855.66 ms (NotFall‐LFOF), 1055.81 ms (Fall‐LFOF), 1971.22 ms (NotFall‐HFOF), and 2187.11 ms (Fall‐HFOF). Reaction time under fatigue (TRTiFatigue and TRTfFatigue) showed a similar trend (*p* < 0.001), with the longest latencies in high‐fear groups (Table 2).

**TABLE 2:** Motor reaction time, cortical arousal, and valence comparisons among experimental groups and the control group.

		Mean ± SD	Confidence interval (CI)	*p*‐value (*ω*)	Pairwise comparison									
					A/B (r)	A/C (r)	A/D (r)	A/E (r)	B/C (r)	B/D (r)	B/E (r)	C/D (r)	C/E (r)	D/E (r)
Simple reaction time (ms)	Control group	352.18 ± 98.21	296.65–615.21	0.007 (0.17)	0.029 (0.28)	—	< 0.001 (0.61)	0.008 (0.58)	0.022 (0.22)	—	< 0.001 (0.78)	0.011 (0.39)	0.014 (0.34)	< 0.001 (0.84)
	NotFall‐LFOF	855.66 ± 103.54	724.19–928.21											
	NotFall‐HFOF	1971.22 ± 109.28	1429.68–2101.45											
	Fall‐LFOF	1055.81 ± 149.57	786.40–1214.23											
	Fall‐HFOF	2187.11 ± 141.04	1876.67–2354.41											
Initial fatigue reaction time (ms)	Control group	578.84 ± 87.98	459.87–625.24	< 0.001 (0.78)	0.021 (0.19)	—	< 0.001 (0.64)	< 0.001 (0.69)	< 0.001 (0.70)	—	< 0.001 (0.75)	< 0.001 (0.69)	< 0.001 (0.54)	< 0.001 (0.88)
	NotFall‐LFOF	1501.84 ± 191.70	1395.44–1785.25											
	NotFall‐HFOF	2310.11 ± 181.85	1865.25–2547.37											
	Fall‐LFOF	1232.98 ± 175.11	913.30–1547.25											
	Fall‐HFOF	2316.04 ± 165.68	1931.27–2547.69											
Final fatigue reaction time (ms)	Control group	417.44 ± 78.61	314.26–509.32	< 0.001 (0.69)	< 0.001 (0.67)	—	< 0.001 (0.56)	< 0.001 (0.51)	< 0.001 (0.51)	—	< 0.001 (0.79)	< 0.001 (0.53)	< 0.001 (0.51)	< 0.001 (0.89)
	NotFall‐LFOF	817.85 ± 119.18	615.19–958.74											
	NotFall‐HFOF	1850.34 ± 158.71	1656.45–2025.86											
	Fall‐LFOF	864.41 ± 121.51	650.77–958.46											
	Fall‐HFOF	1935.37 ± 151.13	1702.74–2102.54											
Cortical arousal^a^	Control group	0.98 ± 0.11	0.97–0.99	0.034 (0.12)	0.029 (0.19)	—	0.024 (0.27)	—	0.032 (0.18)	—	0.031 (0.19)	0.025 (0.24)	—	0.026 (0.24)
	NotFall‐LFOF	0.98 ± 0.19	0.97–0.99											
	NotFall‐HFOF	0.96 ± 0.14	0.95–0.98											
	Fall‐LFOF	0.98 ± 0.17	0.97–0.99											
	Fall‐HFOF	0.96 ± 0.12	0.94–0.97											
Cortical valence^b^	Control group	0.96 ± 0.09	0.95–0.98	< 0.001 (0.71)	< 0.001 (0.81)	—	< 0.001 (0.86)	0.004 (0.67)	< 0.001 (0.79)	—	< 0.001 (0.89)	< 0.001 (0.79)	0.010 (0.68)	< 0.001 (0.84)
	NotFall‐LFOF	0.92 ± 0.11	0.90–0.95											
	NotFall‐HFOF	0.73 ± 0.12	0.70–0.77											
	Fall‐LFOF	0.91 ± 0.09	0.88–0.93											
	Fall‐HFOF	0.71 ± 0.13	0.68–0.79											

*Note:* A, NotFall‐LFOF; B, NotFall‐HFOF; C‐Fall‐LFOF; D‐Fall‐HFOF. Comparative analysis was performed using one‐way ANOVA, considering effect size (*ω*) and significance at *α* ≤ 0.05. Post hoc Tukey pairwise comparisons were conducted considering effect size (*r*) and significance set at *α* ≤ 0.05.

^a^ ΒF3 + ΒF4 + ΒAF3 + ΒAF4)/(αF3 + αF4 + αAF3+ αAF4.

^b^ αF4 − αF3.

Regarding cortical activation, the arousal index (β/α ratio) was slightly reduced in high‐fear groups (0.96 ± 0.14) compared with controls (0.98 ± 0.11) (*p* = 0.034). More pronounced differences appeared in prefrontal valence (αF4/βF4 − αF3/βF3) (*p* < 0.001; *ω* = 0.71), with lower values in NotFall‐HFOF (0.73 ± 0.12) and Fall‐HFOF (0.71 ± 0.13) than in controls (0.96 ± 0.09), indicating a relative dominance of right‐hemispheric activity (Table 2).

Pearson's correlation analysis revealed strong and consistent associations among cortical, motor, and emotional variables. Valence showed a strong negative correlation with all reaction time parameters (*r* > −0.9), indicating that lower valence, meaning greater right‐hemispheric asymmetry, was associated with slower motor responses. Complementarily, the FES‐I score displayed high positive correlations with reaction times (*r* = 0.8–0.9) and negative correlations with both arousal and valence (*r* = −0.7 to −0.9) (Table 3).

**TABLE 3:** Correlation between motor reaction time, cortical arousal, valence, and fear of falling assessed by the Brazilian version of the FES‐I.

		Simple reaction time (ms)	Initial fatigue reaction time (ms)	Final fatigue reaction time (ms)	FES‐I (escore)
Cortical arousal^a^	Control group	−0.325	−0.224	−0.354	−0.378
	NotFall‐LFOF	−0.308	−0.287	−0.396	−0.794
	NotFall‐HFOF	−0.513	−0.547	−0.345	−0.845
	Fall‐LFOF	−0.397	−0.415	−0.361	−0.764
	Fall‐HFOF	−0.689	−0.598	−0.305	−0.831
Cortical valence^b^	Control group	−0.854	−0.865	−0.798	−0.863
	NotFall‐LFOF	−0.886	−0.897	−0.831	−0.801
	NotFall‐HFOF	−0.924	−0.934	−0.921	−0.896
	Fall‐LFOF	−0.914	−0.925	−0.942	−0.798
	Fall‐HFOF	−0.932	−0.915	−0.905	−0.901
FES‐I (escore)	Control group	0.834	0.513	0.868	—
	NotFall‐LFOF	0.414	0.251	0.248	—
	NotFall‐HFOF	0.874	0.664	0.749	—
	Fall‐LFOF	0.421	0.542	0.712	—
	Fall‐HFOF	0.896	0.625	0.654	—

^a^ (ΒF3 + ΒF4 + ΒAF3 + ΒAF4)/(αF3 + αF4 + αAF3+ αAF4).

^b^ (αF4 − αF3); A—NotFall‐LFOF; B‐NotFall‐HFOF; C‐Fall‐LFOF; D‐Fall‐HFOF, no significant correlations were observed (*p* ≤ 0.05). Pearson's correlation test was applied, considering significance at *p* < 0.05. Correlations were interpreted as weak (*r* < 0.30), moderate (*r* = 0.30–0.60), or strong (*r* > 0.60).

## 4 Discussion

The results of this study demonstrate that fear of falling (FoF) directly influences cortical activation and motor performance in older women, independently of previous fall events. The significant prolongation of reaction times and the reduction in cortical valence indicate that fear acts as a neuroemotional modulator, reducing the efficiency of prefrontal–motor circuits. The right‐hemispheric asymmetry observed in participants with high FoF is consistent with negative emotional dominance patterns reported in studies linking anxiety and risk anticipation to altered motor performance [7, 54]. According to the *perceived control of falling model*, FoF and balance confidence interact through perceived control over balance‐threatening situations, which mediates the relation between fear and fall risk.

Neuroimaging studies indicate that the prefrontal cortex plays a crucial role in balance and motor control. High prefrontal–motor coherence during postural reactions has been associated with greater cognitive–motor interference and increased fall risk [55]. Older adults show higher prefrontal activation during balance tasks compared with younger adults, reflecting neural inefficiency. Lower prefrontal activation, conversely, correlates with better postural performance, suggesting that prefrontal asymmetry may represent a compensatory mechanism in aging [56]. Decline in inhibitory control, a key executive function, is also associated with impaired balance and greater fall risk, even in pathological aging such as Alzheimer's disease [57]. Moreover, dual‐task paradigms reveal that brain activity during simultaneous motor–cognitive tasks independently predicts fall profiles in older adults [58], while functional overlap between vestibular and fear‐related networks may modulate postural performance [59].

The progressive slowing of reaction time among participants with high FoF suggests an imbalance between automatic and conscious control of movement. Older adults frequently show compensatory prefrontal hyperactivation in motor and cognitive tasks, consistent with the CRUNCH and STAC‐r models of aging, which propose the recruitment of additional neural resources to maintain performance [60]. However, under conditions of perceived threat, this compensatory mechanism becomes maladaptive, leading to excessive motor monitoring and attentional interference that delay responses [61]. Functional near‐infrared spectroscopy (fNIRS) findings corroborate this pattern, showing that older adults exhibit higher frontal activation during dual tasks as a compensatory attempt to preserve motor control [62].

The association between FoF and reaction time under fatigue reinforces this interpretation. Sustained attention and cortical self‐regulation are essential for daily activities and tend to deteriorate with age, contributing to frailty and increased fall risk [63]. Event‐related potentials and EEG spectral analyses demonstrate that attentional decline and cognitive fatigue are marked by altered oscillatory patterns, such as reduced occipital alpha power and frontal theta variability [64, 65, 66]. Older adults with high FoF also allocate attentional resources to worrisome thoughts and environmental monitoring, which diminishes postural control efficiency [67].

The reduction in prefrontal valence observed in the high‐FoF groups supports the hypothesis of emotional asymmetry. Lower valence (F3–F4) reflects greater right‐hemispheric activity, consistent with patterns associated with negative emotion, risk anticipation, and avoidance behaviours. Although some evidence suggests that older adults exhibit increased left cortical activity compared with younger adults [25], the present findings point to a context‐dependent shift toward right dominance under fear conditions. Age‐related changes in cortical oscillations also affect motor control, as increased theta activity and reduced mu–beta power during balance tasks have been described in older adults [68], and alterations in excitatory–inhibitory balance modulate both cognition and movement [69].

The reduction in cortical arousal (β/α ratio) observed in high‐FoF participants indicates diminished prefrontal engagement and possible top‐down inhibition of motor networks. Age‐related alterations in alpha–gamma synchronization reveal compromised top‐down attentional mechanisms, even when bottom‐up processes remain relatively preserved [70]. The beta/alpha ratio has been proposed as a sensitive index of attentional engagement, predicting lapses in sustained attention [71]. Changes in the excitatory–inhibitory balance, particularly reduced inhibitory activity, contribute to cognitive and motor inefficiency in aging [69].

The approach used to quantify cortical valence and arousal should be considered when interpreting the results. The valence index, calculated as (αF4/βF4) − (αF3/βF3), represents a simplified measure of frontal asymmetry. Although more sophisticated methods, such as log‐transformed alpha power and multi‐electrode indices, are commonly used, this approach is supported by prior applied EEG studies [47, 72, 73, 74, 75] and allows the assessment of functional emotional–cortical dynamics.

The arousal index included bilateral frontal sites (F3, F4, AF3, AF4) to capture broader prefrontal engagement, with AF3/AF4 increasing sensitivity to anterior regions. However, this method provides limited spatial precision and restricts direct comparison with canonical asymmetry models. Thus, findings should be interpreted as reflecting global frontal functional dynamics rather than precise anatomical asymmetry.

Strong negative correlations between valence and reaction time (*r* > −0.9) support the link between emotion and motor performance: greater right‐hemispheric asymmetry was associated with slower responses. Although the magnitude of these correlations is higher than typically reported in the literature, additional analyses reviewed by an independent statistician confirmed their stability, with no evidence of influential outliers. This may reflect the tight coupling between cortical dynamics and motor responses measured within the same task context. Nevertheless, these findings should be interpreted with caution and confirmed in larger samples. Likewise, positive correlations between FES‐I and reaction times, together with negative correlations with arousal and valence, indicate that perceived fear translates into measurable behavioural slowing. Variability in reaction time has been proposed as a marker of cognitive control efficiency, as frontal midline theta power correlates with control demands and response variability [71]. Moreover, emotional processing asymmetry influences response speed, with left‐frontal activation associated with faster emotional reactivity [76].

Recent evidence also highlights the role of beta oscillations in motor planning and inhibition. Age‐related declines in inhibitory control are reflected in altered beta bursts and reduced motor synchronization [77]. The movement‐related beta desynchronization (MRBD) becomes exaggerated with aging, indicating inefficient motor planning and increased cognitive effort during movement [78]. The present findings align with this literature, suggesting that inefficient beta modulation contributes to the observed motor slowing in older women with high FoF.

From a methodological perspective, the use of a consumer‐grade EEG system (EMOTIV EPOC+) should be considered when interpreting the findings. Although this device has been previously validated for research purposes, it presents inherent limitations compared with laboratory‐grade systems, including reduced signal‐to‐noise ratio, lower spatial resolution, and a limited number of electrodes.

Regarding signal quality, preprocessing procedures such as band‐pass filtering and independent component analysis (ICA) were applied to minimize noise and artefacts. However, it is important to acknowledge that consumer‐grade systems may be more susceptible to environmental and physiological artefacts, which can influence spectral estimates.

Additionally, the limited spatial resolution and electrode coverage constrain the precise localization of cortical sources. In this context, the interpretation of “prefrontal” activity should be understood as an approximation based on frontal electrode positioning (F3, F4, AF3, AF4), rather than a definitive mapping of specific cortical subregions. Therefore, the findings should be interpreted in terms of functional frontal dynamics rather than strict anatomical specificity.

Despite these limitations, the EMOTIV EPOC+ offers advantages in ecological validity and feasibility, particularly in studies involving motor tasks and older populations, where more complex systems may be less practical. Thus, the present results should be viewed as evidence of robust functional associations, while future studies using high‐density EEG or multimodal neuroimaging are warranted to refine spatial interpretations.

Additionally, although the sample size was supported by a priori power analysis and allowed the detection of robust effects, smaller effects may not have been identified. Future studies with larger samples are needed to confirm these findings and explore more subtle associations.

Taken together, these results indicate that fear of falling functions as a neuroemotional marker of cortical inefficiency, capable of predicting motor decline before falls occur. This perspective has clinical implications, as traditional prevention programs focused solely on strength and balance may be insufficient without addressing the affective and cortical dimension of movement. Interventions targeting prefrontal symmetry and emotional regulation, such as non‐invasive neuromodulation (tDCS, TMS), immersive virtual reality, and cognitive–motor training, have shown promising results in enhancing motor performance and reducing fall risk.

In summary, fear of falling should be understood not merely as a psychological risk factor but as a neurofunctional mechanism that reshapes the cortical dynamics of aging. The combination of motor slowing and prefrontal hypoactivation observed in this study suggests that fear acts as an emotional brake, altering timing and motor efficiency. Understanding FoF from a neuroemotional framework is therefore essential for developing integrative preventive and therapeutic strategies that promote not only postural stability but also emotional and motor balance in aging.

## Funding

This work was supported by Coordenação de Aperfeiçoamento de Pessoal de Nível superior—Brasil (CAPES)—Finance Code 001 and FAPDF—Fundação de Apoio a Pesquisa do Distrito Federal, Finance Code—00193.00001697/2019–6.

## Ethics Statement

This cross‐sectional analytical study was conducted in a controlled laboratory environment. The research protocol was approved by the Research Ethics Committee of the University of Brasília, Faculty of Ceilândia (approval number 2.109.807). All procedures were performed in accordance with the principles of the Declaration of Helsinki.

## Consent

All participants were informed about the study procedures and objectives and provided written informed consent prior to participation.

## Conflicts of Interest

The authors declare no conflicts of interest.

## Data Availability

The datasets generated and analysed during the current study are available from the corresponding author upon reasonable request.
